# The Cost of the Royal Portsmouth Hospital, Portsmouth

**Published:** 1893-04-22

**Authors:** 


### The Institutional Workshop.

### HOSPITAL ADMINISTRATION.

### THE COST OF THE ROYAL PORTSMOUTH HOSPITAL, PORTSMOUTH.

[The Editor will be glad to have his attention called to similar criticism of other hospitals as it occurs from time to time in the press.]

A full account of this excellent institution, by our special Commissioner, appeared in The Hospital, June 25th, 1892, page 205.

In the course of that report our Commissioner wrote: "Owing to the devoted labours of the present matron, Miss Tillett, and the continuous and intelligent support of the committee, which has the wisdom to vote the necessary funds, all, or nearly all, that was objectionable has been removed. The hospital bears throughout the stamp of being well administered in every department, and we cannot but feel that the committee is to be congratulated in having at the head of its affairs so able and devoted a lady as Miss Tillett undoubtedly it. We gather, however, from recent criticisms in the local press, and from inquiries we have made, that the system of supplying meat by carcase still prevails. It should at once be discontinued, and till it is discontinued the expense per bed for meat must necessarily be higher than under the better and more modern system of buying joints instead of buying carcases. We understand that the steward, though nominally, is not actually under the matron, who therefore lacks complete control over the domestic expenditure. If this affirmation be correct we strongly urge the committee to alter the system at once, so as to give the matron complete control and responsibility over the whole of the domestic management of the institution.

Self Destructive Criticism.

A "Critical Subscriber," who ultimately acknowledged himself to be Mr. Harold R. Pink, recently attacked the committee on the ground of their great extravagance. In his first communication, which was anonymous, he stated that the average cost per bed at Portsmouth was £78 9s. 1d., the average number of daily in-patients for 1891 was 81. In his second letter, which was signed Harold R. Pink, he admits that the average number of beds occupied in 1891 was 102, and not 81 as he first stated; that is to say, he underestimated the number of beds occupied by no less than 21. He further gives the average cost per bed occupied "exclusive of salaries paid and drug accounts" at £44 7s. 8¼d., and not £55 18s. 2d., as given in his anonymous letter, a difference of £11 10s. 5¾d. per bed. Such gross inaccuracies are not only grievously unfair to the hospital and its managers, but must be held to condemn the critic as being quite unreliable. But this is not all, for whereas in the anonymous letter this "critical subscriber" claimed to give the average cost per bed upon every item of expenditure, when writing under his own name he declares it to be impossible "for me to divide details and to place the correct sums" (*i.e.*, in the case of salaries paid and drug accounts!) "against in and out patients." Here then we have evidence that this self-constituted critic did not understand the meaning of the figures which he set himself to criticise with confidence and at great length. There are other points in the letters which prove that the inhabitants of Portsmouth will be well advised to ignore the criticisms of "Critical Subscriber," which considerations of space forbid us to dwell upon.

We must not, however, omit to point out that, to compare the expenditure of a hospital like Winchester, which only had 67 occupied beds, with one like the Royal Portsmouth Hospital, where upwards of 100 beds have been occupied during each of the last three years, is not only unfair, but misleading. No one with a knowledge of the subject would venture upon a comparison of this kind, because experience proves that it must be meaningless as well as useless for all practical purposes. It is always desirable to encourage outspoken criticism based upon knowledge and good feeling. In the circumstances here brought out, no impartial governor could treat the "Critical Subscriber" of the Royal Portsmouth Hospital on such a basis. He is evidently ignorant of his subject, and reckless in regard to the injury which he may inflict upon a hospital, the efficiency of which is so great as to make it an honour to Portsmouth and a credit to the whole country.

The True Facts.

Having dealt with the "Critical Subscriber" aforesaid, we will now endeavour to aid the committee and the inhabitants of Portsmouth by bringing out the actual facts in connection with their expenditure. With the view of doing this effectively, we have analysed the accounts for the last three years, and have compared them with the accounts of other hospitals containing about the same number of occupied beds in different parts of the country. We have no doubt that the figures will prove most interesting to the inhabitants of Portsmouth, and we hope they will lead the local press to persistently advocate the claims of this Royal Hospital, and so to secure that every one who is able to contribute to its funds, will do so without loss of time.

Expenditure of the Royal Portsmouth and Other Hospitals.

As we have said already, no experienced statist with a knowledge of hospital management would venture to compare the expenditure of two hospitals which differed materially in certain crucial respects. In order to make a fair comparison it is essential that the number of beds and the amount of work done should approximate. With this object we have selected the York County Hospital, the Salop Infirmary, Shrewsbury, and the Salford Royal Hospital, have analysed their accounts upon an indentical basis, together with those of the Royal Portsmouth Hospital, and have exhibited the result in the following table. We have made the selection of these three hospitals almost at random, but have thought it well to include a representative type from various sections of the country. The North, South, and Midland districts are thus represented. It will be seen that whereas the number of beds at Portsmouth is 133, and the average number occupied was 104 during the years 1890 and 1891, and 98 during 1892, the number of beds at the three other hospitals averaged 135, whilst the average number occupied was 92 during the years 1890 and 1891. The average number of in-patients treated during the two years in question at the three hospitals was 1,241, and at the Royal Portsmouth Hospital was 1,037 in 1890 and 1891, and 1,073 in 1892.

### Expenditure of Royal Portsmouth and Three other Hospitals Compared.

[York County Hospital, Salop Infirmary, and Salford Royal Hospital.]

| Items Dealt With. | Average for 3 Hospitals for years 1890 & 1891. | Royal Portsmouth, Portsea, and Gosport Hospital. Average for years 1890 & 1891. | Royal Portsmouth, Portsea, and Gosport Hospital. 1892. |
|---|---|---|---|
| Number of beds | 135 | 133 | 133 |
| Daily average number occupied | 92 | 104 | 98 |
| Number of in-patients | 1,241 | 1,037 | 1,073 |
| Number of out-patients | 10,874 | 6,833 | 6,650 |
| Average cost per bed occupied on total expenditure, less extraordinary expenses | £60/5/5 | £60/5/3 | £60/13/11 |
| Average cost per in-patient on total expenditure, less extraordinary expenses | £4/9/5 | £6/0/9 | £5/10/11 |
| Proportion of in-patients to beds occupied | 13 to 1 | 10 to 1 | 11 to 1 |
| | Cost per bed occupied. | Cost per bed occupied. | Cost per bed occupied. |
| | £ s. d. | £ s. d. | £ s. d. |
| Provisions | 20 10 2 | 21 5 1 | 20 12 9 |
| Alcohol | 1 3 4 | 1 2 5 | 1 2 3 |
| Domestic expenses | 9 1 11 | 14 3 11 | 12 19 9 |
| Surgery and dispensary | 8 16 4 | 6 15 2 | 7 12 4 |
| Salaries and wages | 14 10 1 | 11 0 6 | 10 13 7 |
| Pensions | 0 1 5 | | |
| Repairs | 3 14 2 | 3 6 0 | 4 19 2 |
| Incidental expenses | 2 8 0 | 2 12 2 | 2 14 1 |
| Totals | 60 5 5 | 60 5 3 | 60 13 11 |
| Extraordinary expenses | 4 1 1 | 2 19 4 | |

It will be seen from the above table that the management at the Royal Portsmouth, Portsea, and Gosport Hospital has been so continuously economical as to result in an almost identical expenditure during the three years under consideration. Thus, whereas *the average cost per bed occupied* on the total expenditure was £60 5s. 3d. in the years 1890 and 1891, it was only £60 13s. 11d. in 1892. If we take the expenditure in detail we shall find that the slight increase in the cost per bed is caused by the extra sum expended on repairs (a necessary but varying item) in the year 1892. In the year 1892 there was a reduction in the cost per bed for provisions, alcohol, domestic expenses, and salaries and wages as compared with the average for 1890 and 1891. On the other hand, in addition to the increase in repairs, there was a small increase in incidental expenses, and a large one in surgery and dispensary, which is more than accounted for by the increased number of in-patients treated in 1892 as compared with 1890 and 1891. Again, it is highly creditable to the authorities of the Royal Portsmouth Hospital that whereas the average cost per bed occupied on the total expenditure, less extraordinary expenses in 1890 and 1891, was £60 5s. 3d., it averaged £60 5s. 5d. per bed at the three other hospitals during the same years. We have not yet received the reports of the three hospitals for the year 1892, so it is not possible for us to compare the expenditure in that year in the same way. Another important fact which the table brings out is that the number of days during which in-patients were resident at Portsmouth was considerably greater than at the three selected hospitals. This is shown by the fact that the proportion of in-patients to beds occupied in 1890 and 1891 was only ten to each bed at Portsmouth, whereas it averaged thirteen to each bed at the three selected hospitals. The effect of this upon the expenditure is shown by the fact that the average cost per in-patient at Portsmouth during these years was £6 0s. 9d., whereas it was only £4 9s. 5d. at the three selected hospitals, although the average cost per occupied bed was less at Portsmouth than it was at the other institutions. This last fact eloquently testifies to the necessity for exercising great care, out of fulness of knowledge, when making comparisons between the expenditure of various medical institutions.

Another point worthy of notice is that the out-patients treated at the Royal Portsmouth Hospital are materially fewer than the average of those treated at the three other institutions, being 6,833, compared with 10,874 in 1890 and 1891. Until the accounts of the expenditure upon the in and out patient departments at all the hospitals are kept separate it is not possible to accurately ascertain the bearings of these numbers upon the cost per bed occupied. It will be seen that the expenditure upon surgery and dispensary, and salaries and wages is considerably higher at the three selected hospitals than it is at Portsmouth, and that the expenditure on alcohol is also greater. On the other hand, the expenditure per occupied bed upon provisions, domestic expenses, and incidental expenses, is slightly higher on the first and last items, and materially higher on the second item at Portsmouth than it is at the three selected institutions. This difference, so far as provisions are concerned, disappears when the accounts of the Royal Portsmouth Hospital are taken for the year 1892.

### Income of the Royal Portsmouth and Other Hospitals Compared.

We have thought it might add to the interest of this article if we gave a table showing the income from various sources, not only of the Royal Portsmouth but of the three selected hospitals. It will be seen that whereas the average income for the years 1890 and 1891 for dividends and invested property is well-nigh identical, the Royal Portsmouth Hospital receives considerably more from subscriptions, Hospital Saturday and Sunday Funds, patients' payments, and the nursing institution. The Royal Portsmouth, on the other hand received considerably less from donations in 1890 and 1891, though this was rectified in 1892, and Portsmouth is also far behind in the matter of legacies. We may add that the York County Hospital, one of the three selected, received nothing from Hospital Saturday and Sunday Funds, a circumstance to which we beg to direct the urgent attention of the Managers of that institution.

### Income of Royal Portsmouth and other Hospitals Compared.

| — | Average income for three hospitals (York County Hospital, Salop Infirmary, and Salford Royal Hospital) for years 1890 and 1891. | Royal Portsmouth, Portsea, and Gosport Hospital. Average income for years 1890 and 1891. | Royal Portsmouth, Portsea, and Gosport Hospital. Income for 1892. |
|---|---|---|---|
| | £ s. d. | £ s. d. | £ s. d. |
| Dividends and invested properties | 1,764 14 4 | 1,734 6 8 | 1,895 14 5 |
| Subscriptions | 1,516 17 5 | 1,635 18 0 | 1,730 0 0 |
| Donations | 862 12 11 | 456 0 6 | 1,770 12 9 |
| Boxes | 7 4 2 | 19 15 8 | 15 8 5 |
| Legacies | 1,675 15 7 | 1,162 0 0 | 1,236 0 5 |
| Hospital Saturday | 338 15 7 | 450 10 0 | 500 0 0 |
| Hospital Sunday | | 783 15 9 | 932 17 4 |
| Patients' payments | 43 12 2 | 77 14 7 | 103 16 2 |
| Probationers' payments | 97 13 8 | 99 18 4 | 102 17 6 |
| Nursing institution | 144 7 0 | 313 12 6 | 285 10 0 |
| Miscellaneous receipts | 73 16 10 | 90 17 0 | 71 19 10 |
| Total | 6,525 9 4 | 6,824 9 0 | 8,644 16 10 |

